# Full-Azimuth Beam Steering MIMO Antenna Arranged in a Daisy Chain Array Structure

**DOI:** 10.3390/mi11090871

**Published:** 2020-09-19

**Authors:** Kazuhiro Honda, Taiki Fukushima, Koichi Ogawa

**Affiliations:** 1 Graduate School of Engineering, Toyama University, 3190 Gofuku, Toyama 930-8555, Japan; ogawa@eng.u-toyama.ac.jp; 2 Panasonic System Networks R&D Lab. Co., Ltd., Technology Center, 2-5 Akedori, Izumi-ku, Sendai, Miyagi 981-3206, Japan; fukushima.taiki@jp.panasonic.com

**Keywords:** daisy chain multiple-input multiple-output (MIMO) antenna, beam steering array, large-scale MIMO, over-the-air (OTA) testing, Monte Carlo simulation, connected car

## Abstract

This paper presents a multiple-input, multiple-output (MIMO) antenna system with the ability to perform full-azimuth beam steering, and with the aim of realizing greater than 20 Gbps vehicular communications. The MIMO antenna described in this paper comprises 64 elements arranged in a daisy chain array structure, where 32 subarrays are formed by pairing elements in each subarray; the antenna yields 32 independent subchannels for MIMO transmission, and covers all communication targets regardless of their position relative to the array. Analytical results reveal that the proposed antenna system can provide a channel capacity of more than 200 bits/s/Hz at a signal-to-noise power ratio (SNR) of 30 dB over the whole azimuth, which is equivalent to 20 Gbps for a bandwidth of 100 MHz. This remarkably high channel capacity is shown to be due to two significant factors; the improved directivity created by the optimum in-phase excitation and the low correlation between the subarrays due to the orthogonal alignment of the array with respect to the incident waves. Over-the-air (OTA) experiments confirm the increase in channel capacity; the proposed antenna can maintain a constant transmission rate over all azimuth angles.

## 1. Introduction

One of the most straightforward approaches for improving the capacity of multiple-input multiple-output (MIMO) systems is to use a large number of antenna elements. The concept of large-scale MIMO or massive MIMO systems with more than 100 antenna elements has been proposed for both fifth and sixth generation (5G and 6G) mobile communications [1,2].

**Figure 1:**
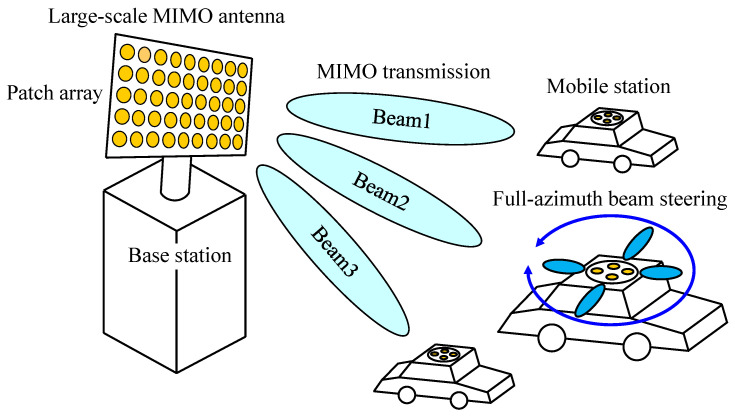
Conceptual illustration of a massive MIMO system.

Figure 1 shows a conceptual illustration of a massive MIMO system. In massive MIMO systems, investigations are mostly based on the development of a large-scale antenna at a base station, in which a planar array antenna with a number of patch antennas arranged in a two-dimensional manner on a column-row alignment basis is commonly developed [3,4,5,6], as depicted in Figure 1. The primary objective of these antennas is, using a beam forming technique, to illuminate respective mobile stations distributed in a specific confined region of the service area in a cell, known as a hot-spot. Thus, a large-scale MIMO antenna used for massive MIMO systems is capable of communicating with mobile stations in the hemispherical spatial region perpendicular to the surface of the patch array. With regard to a mobile station antenna, on the other hand, one of the most important performance goals in MIMO systems is the ability to communicate with the target over the full azimuth, i.e., full-azimuth beam steering, as illustrated in Figure 1.

One of the possible solutions to fulfilling this requirement is the use of a circular array in which a number of patch antennas are arranged in a cylindrical manner [7,8]. However, there is a certain drawback to this configuration in that each patch antenna radiates in a direction normal to the surface of the patch, resulting in a situation in which not all of the radiation beams contribute to MIMO spatial multiplexing when an incident wave comes from a particular azimuthal direction. Consequently, we cannot obtain full-rank channel matrices corresponding to the number of patches due to the limited availability of the received signals, which leads to a reduction in channel capacity.

There has been a great deal of interest in the development of connected car systems with greater than a gigabit transmission rates [9]. It is anticipated that the MIMO antennas implemented in automobiles will be used in systems with a relatively small cell radius involving a street microcell [10]. In such an environment, the various objects in the surrounding area—such as trees, signboards, buildings, and numerous vehicles—result in complex radio propagation phenomena. In a street microcell environment, it is known that the spatial angular power spectrum (APS) is small compared with that in the conventional macrocell counterpart [11]. This is because the incident waves from these scatterers are not uniformly distributed, but occur in clusters, with strong contributions from a few directions. In addition, the radiation pattern of a vehicular antenna changes greatly due to the mutual electromagnetic coupling between the antenna and the dynamic motion of the car, especially when it turns right or left at an intersection. This indicates that the mutual influence of the incident waves and the car’s antennas could result in a complicated MIMO channel response.

Based on the above-mentioned phenomena, the characteristics of a vehicular MIMO antenna are summarized as follows:

(1) Anticipated changes in received power when the position of the car changes relative to the incident waves.
(2) Increase in spatial fading correlation between the array branches due to the narrow APS.
(3) Changes in both the received power and correlation, which may be encountered at the same time, as the car moves in different directions relative to the incident waves.
(4) A possible increase or decrease in the MIMO channel capacity caused by (1), (2), or (3).

These phenomena may occur simultaneously in an actual connected car system. Hence, all the solutions with regard to the above issues must be integrated into one unit in the developed MIMO antenna. Considering this technical background, we have developed new technologies in an ongoing project with the following objectives:

(A) Development of higher order MIMO arrays, such as 8 × 8 [12], 16 × 16 [13], and 32 × 32 [14,15] MIMO antenna systems, suitable for implementation in an automobile.
(B) Development of radiation pattern steering capability to achieve a large signal-to-noise power ratio (SNR) that can direct the peak radiation toward the communication target.
(C) Realization of low correlation between the MIMO channels established by the orthogonal relationship between the array alignment and the incident waves over the full azimuth.
(D) Development of an angle of arrival (AOA) estimation antenna that obtains bearing information from radio waves incident on the MIMO antenna, using an RF-based interferometric monopulse technique with reduced hardware complexity.

To realize these objectives simultaneously, we are currently developing a 32 × 32 MIMO antenna system that utilizes circular array beam steering technology. Great emphasis is placed on a way of achieving a large-scale vehicle borne MIMO antenna that provides full-azimuth coverage. The new array configuration proposed in this paper yields the full availability of the received signals, which results in a channel capacity of several tens of gigabits owing to the effective operation of the MIMO multiplexing transmission.

This paper, which is part of the extensive R&D activities we have performed so far in this field, is devoted to a comprehensive description of the development work undertaken for a 32 × 32 MIMO antenna system, including Monte Carlo simulations and over-the-air (OTA) testing to investigate how the developed 32 × 32 MIMO array performs in a propagation environment where it is anticipated the forthcoming 5G and 6G connected car systems will be used.

In order to tackle the four challenges—(A), (B), (C), and (D), listed above—we start by describing the methodology and the basic characteristics of MIMO antennas. We first present a brief explanation of the configuration of the whole antenna system. Studies on the AOA antenna are presented in separate papers [16,17].

## 2. New Concept of a Large-Scale MIMO Antenna

**Figure 2:**
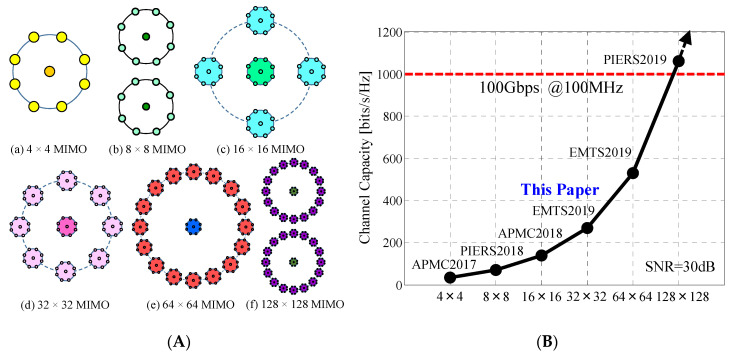
A big challenge toward 100 Gbps channel capacity. (**A**) A family of daisy chain MIMO antennas. (**B**) History of the development.

In Figure 2 an illustrated overview of our ongoing project, conducting research and development work toward a full-azimuth beam steering MIMO array, is shown [18]. We have taken on the big challenge of achieving a 100 Gbps channel capacity on a moving vehicle for the forthcoming sixth-generation (6G) mobile communications. To this end, we have devised effective means of constructing a large-scale MIMO array antenna, with distinct features that cannot be achieved using previous technologies commonly aimed at enhancing the channel capacity at a base station; the objective of our project is accomplished by the emergence of a new technology named “Daisy Chain MIMO Antenna”.

Figure 2A shows a family of daisy chain MIMO antennas, which comprises a plurality of 4 × 4 MIMO antennas, as a basic component module, arranged in a variety of two-dimensional geometrical configurations. The term “4 × 4 MIMO” means that the number of elements at the base station (former number) is four and that at the mobile station (latter number) is four. Therefore, we have four subchannels for MIMO transmission in this case. This new approach enables us to construct a high-order MIMO antenna with great ease since the same fabrication process used to fabricate 4 × 4 MIMO antennas is used for each configuration. For example, as shown in Figure 2A, the 32 × 32 MIMO antenna has a circular array of eight 4 × 4 MIMO antennas, resembling a daisy chain with eight pink flowers, forming a 32 × 32 MIMO array as a whole. Hence, we have named this type of antenna “Daisy Chain MIMO Antenna”.

Figure 2B shows the history of the development of the daisy chain MIMO antenna. The figure illustrates the channel capacity achieved using the different size arrays, together with the abbreviated name of the international conference where the corresponding paper was presented. As shown in the figure, we have accomplished a twofold increase in channel capacity as the size of the array doubles. Consequently, we have achieved a channel capacity greater than 100 Gbps for a bandwidth of 100 MHz by means of a 128 × 128 MIMO array, as indicated in Figure 2B. Among the various configurations categorized in the family in Figure 2A, this paper focuses on a detailed description of the design and performance of a daisy chain 32 × 32 MIMO antenna.

## 3. Daisy Chain 32 × 32 Multiple-Input Multiple-Output (MIMO) Antenna

**Figure 3:**
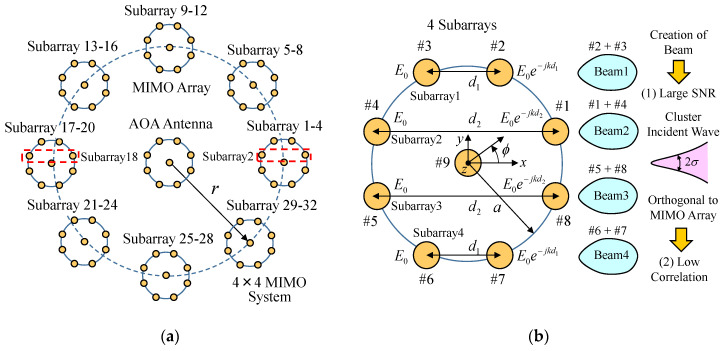
Beam steering MIMO antenna arranged in a daisy chain array structure. (**a**) The whole structure of a 32 × 32 MIMO system. (**b**) 4 × 4 MIMO system.

Figure 3 shows the configuration of a beam steering MIMO antenna arranged in a daisy chain array structure [14]. Figure 3a shows the whole structure of the MIMO antenna system. Figure 3b shows a 4 × 4 MIMO circular array [19], which is used as a component module to form the 32 × 32 MIMO array. As shown in Figure 3a, the developed MIMO antenna comprises 64 elements arranged in a daisy chain array structure, forming a dual-ring configuration; one ring is formed by the 4 × 4 MIMO array, and the other ring is constructed from eight sets of 4 × 4 MIMO arrays, arranged in a circle with radius *r* at equal angular intervals of 45°, constructing the 32 × 32 MIMO array as a whole.

32 subarrays are created by pairing elements in each 4 × 4 MIMO array; the antenna yields 32 independent subchannels for MIMO transmission, and covers all communication targets regardless of their position relative to the array, as mentioned below. As illustrated in Figure 3b, four pairs of the eight elements form four subarrays (Elements #2–3, #1–4, #5–8, and #6–7). All the elements are half-wavelength dipole antennas. These subarrays create four independent radiation patterns (Beam1–Beam4), each of which acts as a subchannel for the MIMO array by enabling excitation of the four subarrays which achieves an in-phase state toward the target direction of communication. As a result, the peak gain of the beam is larger than that of an ordinary dipole antenna, which results in a large SNR.

In Figure 3b, *k* represents the wave number, *d*_1_ and *d*_2_ denote the distance between Elements #2 and #3, and Elements #1 and #4, respectively. *E*_0_ signifies the amplitude of the electric field, and *j* indicates the complex unit. Using these parameters, the excitation conditions for the four subarrays to establish an in-phase state for the formation of a beam toward the communication target are described in Section 5.

Another unique feature of the 4 × 4 MIMO antenna is the realization of low correlation. When an incident wave with a narrow APS arrives from the right side of the figure, the four subarrays are arranged perpendicular to the incident wave. In general, in a cluster propagation environment, a MIMO array parallel to an incident wave results in high correlation between the branches [20]. In contrast, a MIMO array orthogonal to an incident wave yields low correlation. Hence, the orthogonal arrangement between the subarray and the cluster incident wave, as shown in Figure 3b, results in a smaller correlation coefficient, which is beneficial for the enhancement of the MIMO channel capacity, together with the creation of a large SNR due to the four subarrays mentioned above.

**Figure 4:**
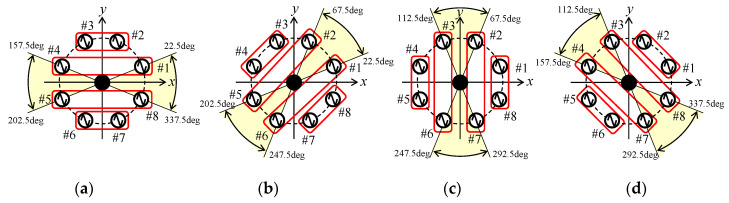
Combinations of the subarrays. (**a**) Combination1. (**b**) Combination2. (**c**) Combination3. (**d**) Combination4.

In Figure 3b, as the eight elements form a circular arrangement with 45° intervals, the subarrays can be rotated every 45° in the azimuth plane. Figure 4 shows the four combinations of subarrays according to the angle of the incident wave [19]. The yellow zone shows the angular range of the incident wave applicable to MIMO communications. Therefore, when an automobile turns right or left at an intersection, which results in a situation where an incident wave arrives from other azimuth angles, we can select other appropriate combinations from among the possible combinations of subarrays. This function can also be applied effectively to the 32 × 32 MIMO beam steering array antenna, by performing synchronized switching of all the 32 subarray beams. This unique feature allows the SNR of the 32 subarray beams to be large, and simultaneously, the correlation to be small, which keeps the channel capacity in a high-bit rate condition, even with considerable dynamic motion of the car.

**Figure 5:**
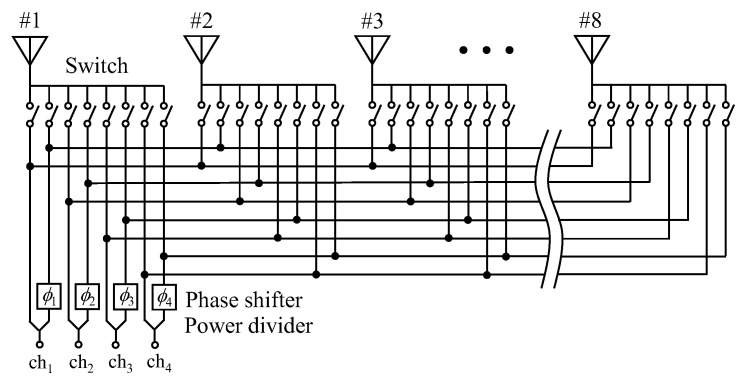
Beam forming network for full-azimuth steering.

Figure 5 shows a schematic diagram of a beam forming network for full-azimuth steering, which comprises switches, phase shifters, and power dividers [15]. Using this network, the four combinations of subarrays, as shown in Figure 4, can be chosen according to the angle of the incident wave to deliver signals to Elements #1–8 for achieving an in-phase state between a pair of elements.

## 4. Formulation of Monte Carlo Simulation

**Figure 6:**
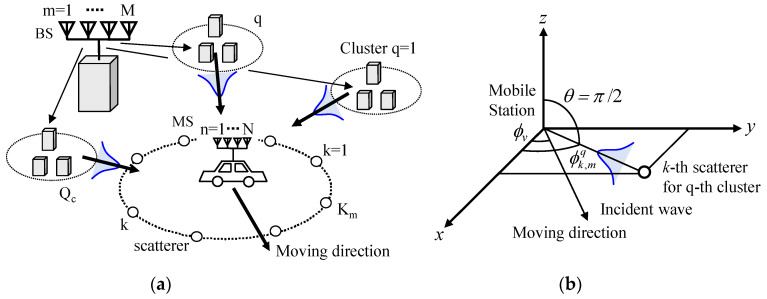
Channel model used for performing the Monte Carlo simulation. (**a**) Cluster channel model of *M* × *N* MIMO. (**b**) Coordinates of the *k*-th scatterer.

This section is devoted to the formulation of a Monte Carlo simulation used to analyze the channel responses of the daisy chain MIMO antenna. As shown in Figure 6a, the concept of clusters [21] is employed to simulate a set of discrete waves with narrow APS arriving at a mobile station (MS). In Figure 6, the bell-shaped blue curves represent a cluster incident wave. We have developed Monte Carlo simulation software using MATLAB, in which a cluster channel model suitable for simulating a large-scale MIMO antenna at the mobile side is formulated. The developed software is an improved version of the channel modeling used for simulating handset adaptive and MIMO arrays with a uniform azimuthal APS [22,23].

In order to simulate a small cell scenario, each cluster is assumed to have a large number of arrival paths, ensuring the full-rank status of a channel matrix created by a large-scale MIMO antenna. Here, the term ‘path’ is used to represent independent subchannels for MIMO transmission which are created by reflecting or diffraction points from surrounding objects in a cell, such as buildings, trees, and cars. Hence, in an actual cellular system, the number of paths may change greatly, depending on the propagation environment arising from congested urban or non-crowded rural areas. Considering this fact, the prime objective of the channel model used in this paper is to assess the theoretical limitation of the channel capacity achieved by the daisy chain MIMO antenna when a sufficiently large number of paths are available in a cell where a relevant antenna operates.

*M* base station (BS) antennas create a set of *Q_c_* clusters, each of which comprises *M* uncorrelated waves, forming *K_m_* scatterers surrounding *N* MS antennas. Thus, the *M* uncorrelated waves are subject to an independent and identically distributed (i.i.d) complex Gaussian process. Furthermore, the correlation characteristics of the BS and MS sides are taken to be independent of each other, based on the Kronecker assumption [24]. MS antennas are assumed to be surrounded by *K_m_* scatterers, created by *Q_c_* clusters. The *K_m_* scatterers belonging to the *q*-th cluster are Gaussian distributed in azimuth in two-dimensional coordinates. Figure 6b depicts the coordinates of the *k*-th scatterer, in which the MS is moving toward the azimuth direction, *ϕ_v_*. Using the above-mentioned channel modeling, we carried out a Monte Carlo simulation according to the following procedure:

### 4.1. Step 1: Generation of K_m_-Path

**Figure 7:**
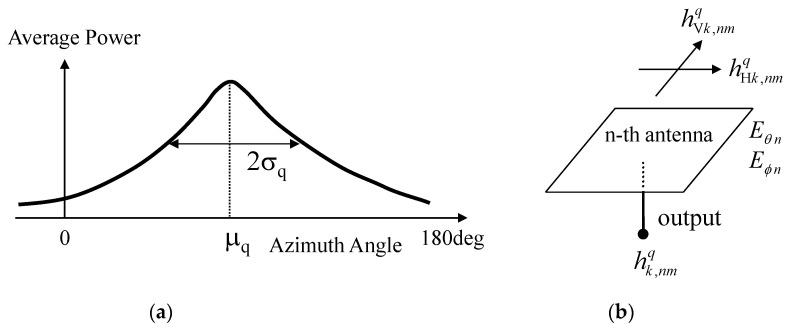
Incident wave model for the Monte Carlo simulation. (**a**) Gaussian incident wave in azimuth. (**b**) Two polarization components.

The multipath property is modeled by the angular power spectrum. The spectrum of the Gaussian distribution for the *q*-th cluster is shown in Figure 7a, and given below.

(1) $$P_{H\theta }(\frac{\pi }{2},\phi )=\frac{XPR}{1+XPR}A_{H\theta }\exp\left[\frac{{\left\{\phi -\left(\frac{\pi }{2}-\mu_{q}\right)\right\}}^{2}}{2\sigma_{q}{}^{2}}\right]$$

(2) $$P_{H\phi }(\frac{\pi }{2},\phi )=\frac{1}{XPR}P_{H\theta }(\frac{\pi }{2},\phi )$$

where *µ_q_* is the azimuth angle of the mean of the incident wave, *σ_q_* is the standard deviation of the power spectrum, and XPR is the cross-polarization power ratio, given as the propagation parameter, and is assumed to be the average value [25]. The channel responses of the signals for a particular snapshot of fading are generated using random numbers, which ensures orthogonality between subchannels for the purpose of ideal MIMO transmission with a full-rank channel matrix.

### 4.2. Step 2: Generation of Polarization and Summation of All Clusters

It is assumed that the *k*-th path for the *q*-th cluster has a transfer function with vertical and horizontal components, as shown in Figure 7b. The vertical and horizontal components of the transfer function of the *k*-th path for the *q*-th cluster at the *n*-th antenna are given by

(3) $$h{\;}_{Vk,nm}^{q}\;=\;\sqrt{\frac{XPR}{1+XPR}}h_{k,m}^{q}\;E_{\theta \;n}(\frac{\pi }{2},\phi {\;}_{k,m}^{q})\exp\left(j\varphi {\;}_{Vk,m}^{q}\right)$$

(4) $$h{\;}_{Hk,nm}^{q}\;=\;\sqrt{\frac{1}{1+XPR}}h_{k,m}^{q}\;E_{\phi \;n}(\frac{\pi }{2},\phi {\;}_{k,m}^{q})\exp\left(j\varphi {\;}_{Hk,m}^{q}\right)$$

where *E_θn_* (*π*/2, *ϕ^q^_k,m_*) and *E_ϕn_* (*π*/2, *ϕ^q^_k,m_*) are the complex electric field directivities of the *n*-th antenna element in the *xy*-plane for the *θ* and *ϕ* components, respectively, which are defined at the origin of the coordinates. *h^q^_k,m_* represents the equivalent amplitude of the incident waves and can be set to an arbitrary value; thus, it is assumed to have unity amplitude. XPR is the cross-polarization power ratio. The phases of the vertical and horizontal polarization components, *φ^q^*_V*k,m*_ and *φ^q^*_H*k,m*_, are independent of each other, and are uniformly distributed from 0 to 2*π*. For each path, the two polarization components are combined with reference to the schematic diagram shown in Figure 7b, as the complex sum of the vertical and horizontal components, and we have

(5) $$h{\;}_{k,nm}^{q}\;=\;h{\;}_{Vk,nm}^{q}+h{\;}_{Hk,nm}^{q}$$

where the transfer function of the *k*-th path is evaluated by summing all the cluster responses, and is given by

(6) $$h_{k,nm}\;=\;\sum_{q=1}^{Q_{c}}h_{k,nm}^{q}$$

### 4.3. Step 3: Generation of the Resultant Channel Response

Using the transfer function represented by Equation (6), the resultant channel response at the *n*-th antenna is calculated using the equation

(7) $$h_{nm}\;=\;\sum_{k=1}^{K_{m}}h_{k,nm}\exp\;\left\{j\frac{2\pi d}{\lambda }\;\cos(\phi {\;}_{k,m}\;-\;\phi {\;}_{v})\right\}$$

where *λ* is the wavelength in free space, and *d* is the distance traveled by a mobile station moving toward the azimuth direction *ϕ_v_*, as shown in Figure 6b. This scheme is applied repeatedly to generate the following channel response matrix at the *s*-th snapshot

(8) $$H_{s}=\left[h_{nm}\right]=\left[\begin{array}{cccc} h_{11} & h_{12} & \cdots & h_{1m}\\ h_{21} & h_{22} & \cdots & h_{2m}\\ \vdots & \vdots & \ddots & \vdots \\ h_{n1} & h_{n2} & \cdots & h_{\mathrm{NM}} \end{array}\right]$$

The distance traveled by the mobile station between two successive points, Δ*d*, in the simulation is changed according to the angular spread of the incident waves, because a narrow spread results in a long fading period. For each value of *d*, fading is assumed to be quasi-static, i.e., the channel response is kept constant.

The complex correlation coefficient between two channel responses is defined as

(9) $$\rho_{c}=\frac{\langle h_{1}{}^{*}h_{2}\rangle }{\sqrt{\langle h_{1}{}^{*}h_{1}\rangle }\sqrt{\langle h_{2}{}^{*}h_{2}\rangle }}$$

where *h*_1_ and *h*_2_ represent the two distinct channel responses in the matrix of Equation (8), and <*X*> denotes the ensemble average of *X* where Y* represents the complex conjugate of *Y*. Using this definition, the absolute value of the complex correlation coefficient can be obtained from

(10) $$\rho_{a}=\left|\rho_{c}\right|$$

Here, the absolute value of the complex correlation coefficient *ρ_a_* in Equation (10) is used as a general description of the correlation behavior of the MIMO antenna performance throughout this paper, as mentioned in Section 5.

### 4.4. Step 4: Evaluation of the Channel Capacity

The eigenvalues and eigenvectors are obtained using the following SVD (singular value decomposition) operation

(11) $$H_{s}=U_{s}D_{s}V_{s}^{H}$$

(12) $$U_{s}=\left[e_{r1},\cdots ,e_{rL}\right]$$

(13) $$V_{s}=\left[e_{t1},\cdots ,e_{tL}\right]$$

(14) $$D_{s}=\mathrm{diag}\left[\sqrt{\lambda_{1}},\cdots ,\sqrt{\lambda_{L}}\right]$$

where *L* = min (*N*, *M*). **U*_s_*** and **V***_s_* are the singular vectors of the receiving (mobile station) and transmitting (base station) antennas, respectively. **D*_s_*** represents the singular values, where *λ_i_* denotes the eigenvalue of the *i*-th subchannel for spatial multiplexing transmission in the MIMO system. Using these eigenvalues, the instantaneous channel capacity and its average value are finally evaluated by

(15) $$C_{s}=\sum_{i=1}^{L}\log_{2}\left(1+\frac{\gamma \;\lambda_{i}}{M}\right)$$

(16) $$\bar{C}=\frac{1}{S}\sum_{s=1}^{S}C_{s}\;(s=1\cdots S)$$

where *γ* is the input SNR (signal-to-noise power ratio), defined as the SNR for each incident wave when an isotropic antenna is used for receiving the incident wave, permitting the performance of the antenna elements used in the MIMO array to be included in the simulation results.

## 5. Theoretical Investigation

### 5.1. Design of 4 × 4 MIMO Antenna

As described in Section 3, the 4 × 4 MIMO antenna depicted in Figure 3b is used as a basic component module or a fundamental functional unit cell to construct the 32 × 32 MIMO antenna array. Hence, in the first step of our investigation, we considered the design of the 4 × 4 MIMO antenna [19].

Two of the eight antenna elements arranged in the circle form a subarray that receives a subchannel of the MIMO communication, where the number of subarrays in the 4 × 4 MIMO antenna is four. The radius of the circle, *a*, was set to 4.9 cm so that the distance between adjacent elements (*d*_1_ shown in Figure 3b) was a quarter-wavelength at 2 GHz. In this case, the directivity of Subarray1 is expected to have a cardioid radiation pattern in the absence of mutual electromagnetic coupling between antenna elements.

**Figure 8:**
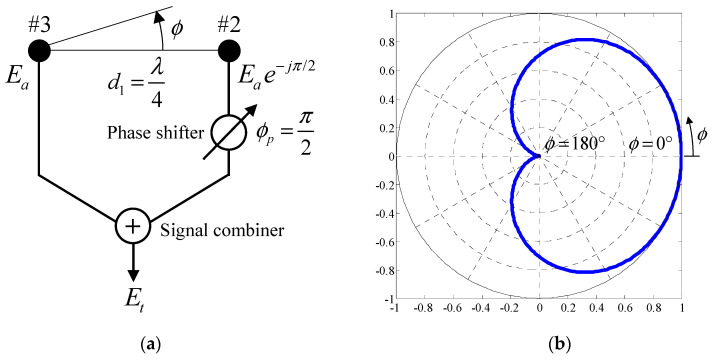
Principle of the cardioid radiation pattern created by two isotropic point sources. (**a**) Configuration of the array. (**b**) Cardioid radiation pattern.

Figure 8 shows the fundamental principle of the cardioid radiation pattern created by two isotropic point sources corresponding to Elements #2 and #3 in Figure 3b, in which there is no electromagnetic mutual coupling between the two sources. As shown in Figure 8a, the distance between the two sources is set to *d*_1_ = *λ*/4. Furthermore, the received signal from Element #2 is delayed by *ϕ_p_* = *π*/2 using a phase shifter. The total received signal, *E_t_*, is given by summing the received signals from Elements #2 and #3 using a signal combiner, and calculated from the equation

(17) $$E_{t}=E_{a}\left[1+\exp\left\{j\frac{\pi }{2}\left(\cos\phi -1\right)\right\}\right]$$

where *ϕ* represents the azimuthal angle defined in Figure 8. *E_a_* denotes the amplitude of excitation of each element. Using this circuit topology shown in Figure 8a, an actual signal processing unit was fabricated, as described in Section 6 (see Figure 18a).

Figure 8b illustrates the radiation pattern normalized to its peak value, calculated by the absolute value of Equation (17). As can be seen from Figure 8b, the radiation pattern directs its maximum towards *ϕ* = 0° and exhibits a deep null towards *ϕ* = 180°. Therefore, the array works as a phased array antenna that yields a strong radiation intensity in the direction of the line between the two sources.

When the subarrays described in Figure 3b are investigated, the electromagnetic mutual coupling between the dipole antenna elements must be considered. The signals received by two elements are multiplied by the weight functions and phase control between the two elements. In Subarray1 in Figure 3b, the weight functions of each element (*w*_2_ and *w*_3_) and the phase shift value (*τ*_2_) are optimized by Equation (18) using the two electric field directivities (*E*_2_ and *E*_3_) of the angle of the incident wave (*ϕ*) when the antenna elements are excited individually.

(18) $$E_{s1}\left(\phi \right)=\frac{w_{2}E_{2}\left(\phi \right)e^{-j\tau_{2}}+w_{3}E_{3}\left(\phi \right)}{\sqrt{60\left(P_{2}+P_{3}\right)}}$$

where *P*_2_ and *P*_3_ are the input power at the feed point of Elements #2 and #3, respectively. The phase of one element shifts to an in-phase state with respect to the other element at the angle of the incident wave in consideration of the mutual electromagnetic coupling. The weight functions are determined in such a way that the gain in the communication direction of the combined radiation pattern yields the maximum value. In Subarray2, the same procedure is applied to optimize the radiation pattern. In Section 6, we will introduce a microwave circuit for realizing weight functions in an experimental way (see Figure 18a).

**Figure 9:**
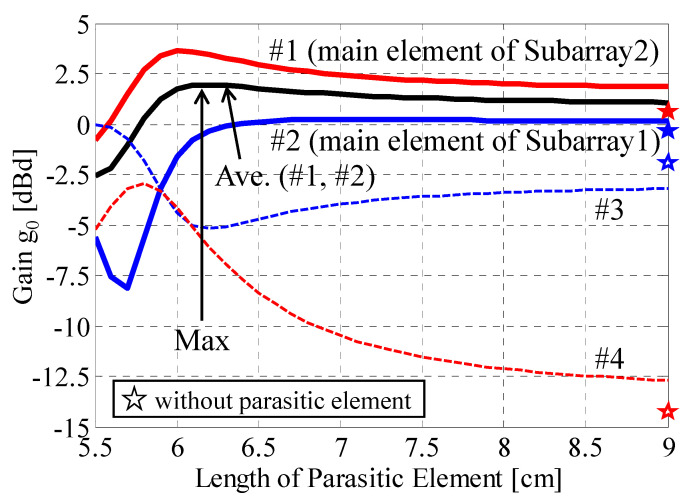
Radiation gain of each element.

Since the maximum radiation gain of each element is approximately 0 dBd, in order to further enhance the radiation gain, we examined placing a parasitic or unexcited element, Element #9 shown in Figure 3b at the center of the circle. Figure 9 shows the radiation gain at 0° as a function of the length of the parasitic element [19]. The parasitic Element #9 works as a reflector or director, similar to a Yagi–Uda array antenna, when its length is long or short, respectively. The blue and red solid lines indicate the radiation gain at 0° of Elements #2 and #1, respectively, which are the most influential elements on the directivities of Subarray1 and Subarray2, when the incident wave comes from 0°. The black line shows the average value of the radiation gain of Elements #1 and #2 at 0°. The star marks plotted on the right axis represent the radiation gains of each element (#1, #2, #3, and #4) when the parasitic element #9 was not in place. Figure 9 shows that when the length of the parasitic element is shorter than a half wavelength (0.5 λ = 7.5 cm), the radiation gains of Elements #1 and #2 are enhanced. When the length of the parasitic element is set to 6.2 cm (0.41 λ), the average radiation gain of Elements #1 and #2 at 0° is at its maximum value.

The excitation conditions for Subarrays 1–4 are summarized in Table 1, which is obtained by searching for the optimum amplitude and phase from among the possible weight functions of each element using Equation (18). Furthermore, Element #9 is used as a parasitic or non-exciting element to enhance the directivity. From the analytical results given in Figure 9, the length of the parasitic element is set to 6.2 cm (0.41 *λ*). As shown in Figure 4, the subarray combinations are the same when the angle of the incident wave is 0° and 180°. However, the optimized weight function and phase shift values of each element at *ϕ* = 0° are different from those at *ϕ* = 180°. In Combination1, since the subarray has a symmetrical configuration with respect to the *y*-axis, the optimum value of element #2 at *ϕ* = 0° is that of #3 at *ϕ* = 180°, whereas the optimum value of #3 at *ϕ* = 0° is that of #2 at *ϕ* = 180°. Thus, we can select other appropriate combinations from among the possible combinations of subarrays.

**Figure 10:**
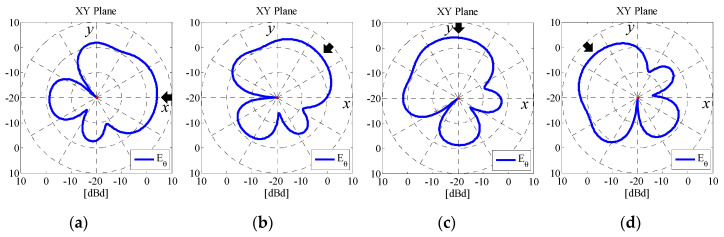
Radiation pattern of Subarray2 when the 4 × 4 MIMO antenna is used. (**a**) *ϕ* = 0 deg (Combination1). (**b**) *ϕ* = 45 deg (Combination2). (**c**) *ϕ* = 90 deg (Combination3). (**d**) *ϕ* = 135 deg (Combination4).

Figure 10 shows the *θ*-polarized radiation patterns in the *xy*-plane of Subarray2 as a function of the angle of the incident wave [19]. The angle of the incident wave is changed from 0° to 135° in 45° intervals. The black arrows in the figure indicate the angle of the incident wave. The radius of the circle, *a*, is set to 4.9 cm. The frequency used in the analysis is 2 GHz. As shown in Figure 10, the radiation patterns vary depending on the angle of the incident wave, which is in accordance with the subarray combination, as illustrated in Figure 4. Furthermore, a radiation gain of 4.1 dBd at the angle of the incident wave is achieved and is larger than an ordinary dipole antenna, which results in a large SNR. The directivities of the other three subarrays also control the direction of the beam. Therefore, high signal levels can be maintained even though the direction of the incoming wave varies due to the dynamic motion of the automobile in connected car applications.

### 5.2. Performance Evaluation of 32 × 32 MIMO Antenna

Table 2 lists the analytical conditions used for performing the Monte Carlo simulation. The number of clusters and number of scatterers were set to 1 and 30, respectively. The number of samples was assumed to be 5000. In the Monte Carlo simulation, the mobile station was moved by a distance of 100*λ* to 1000*λ*, which was changed depending on the angular spread, with the distance between two successive points in the simulation being Δ*d* = 0.02*λ* to 0.2*λ*.

One of the most important considerations in the development of the 32 × 32 MIMO antenna is that the desired directivities of the 32 subarrays are varied via electromagnetic (EM) coupling due to the proximity of the antenna elements. In particular, the radiation patterns of the 16 elements that are closest to the AOA antenna located at the center of the circle, exhibit a significant reduction. This fact allows the channel capacity to be reduced because the directivity of the subarray, which is mainly composed of these elements, cannot lead the beam in the direction of the incoming wave.

**Figure 11:**
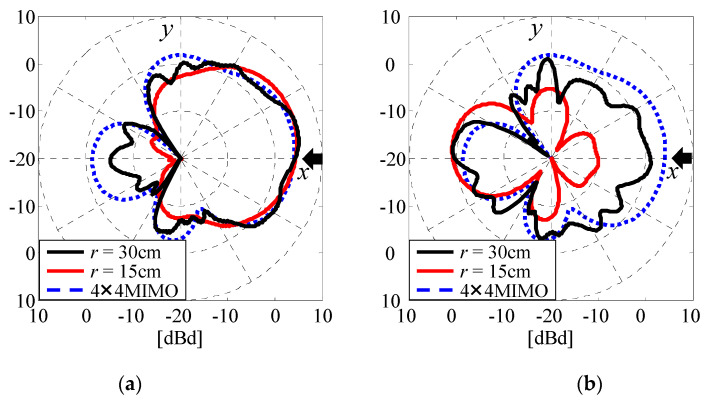
Radiation patterns of Subarray2 and Subarray18 when the 32 × 32 MIMO antenna is used. (**a**) Subarray2. (**b**) Subarray18.

Figure 11 shows the radiation patterns of two subarrays, Subarray2 and Subarray18, as functions of the radius *r* of the array when the angle of the incident wave *ϕ* is 0° [14]. These two subarrays are depicted in Figure 3a by the two red broken rectangles. The excitation conditions listed in Table 1 are used for all the subarray groups in the 32 × 32 MIMO antenna.

In Figure 11, the black and red solid lines indicate the radiation patterns at *r* = 30 cm and *r* = 15 cm, respectively. The blue broken line indicates the radiation pattern of the 4 × 4 MIMO array without EM coupling caused by other 4 × 4 MIMO arrays [19]. The black arrows in the figure show the angle of the incident wave. As shown in Figure 11, the radiation gains at an incident-wave angle of 0° for Subarray2 is constant regardless of the radius *r*, while the radiation gain at *ϕ* = 0° for the 32 × 32 MIMO array agrees well with that for the 4 × 4 MIMO array. This is because the radiation beam from Subarray2 is directed to the outside of the whole antenna system and is less affected by the EM coupling caused by the other 4 × 4 MIMO arrays.

In contrast, the radiation gains at *ϕ* = 0° for Subarray18 are significantly degraded by the EM coupling when *r* = 15 cm. This is due to the fact that Subarray18 directs its radiation beam toward the AOA antenna and is significantly affected by EM coupling with the AOA antenna. However, the radiation gain at *r* = 30 cm is improved considerably and is close to that of the 4 × 4 MIMO array. These results show that the radiation gain at the angle of the incident wave increases with increasing radius due to smaller EM coupling.

The final target of our study is to obtain transmission rates greater than a gigabit to ensure the success of these arrays in upcoming connected car systems. Hence, to investigate the optimum radius for this purpose, the channel capacity is calculated through Monte Carlo simulations, with a single cluster Gaussian spectrum coming from azimuth angle *ϕ*, as depicted in Figure 3b.

**Figure 12:**
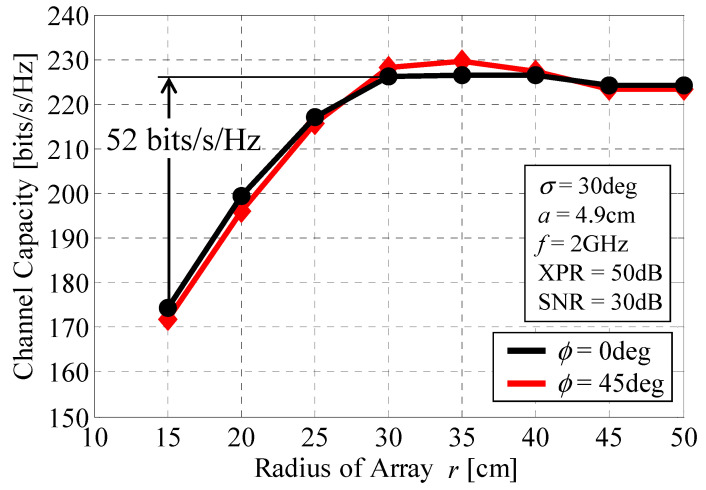
Channel capacity as a function of the radius of the array.

Figure 12 shows the average channel capacity, calculated from Equation (16), as a function of the radius *r* at 2 GHz [14]. The black and red curves correspond to incident-wave angles *ϕ* of 0° and 45°. Half-wavelength dipole antennas are used for the 64 elements. The radius of the 4 × 4 MIMO antenna *a* is set to 4.9 cm. The cross-polarization power ratio, XPR, is set to 50 dB, which is equivalent to a vertically polarized propagation environment. The standard deviation of the incident wave *σ* (*σ*_1_ for the cluster1 in Figure 7a) is set to 30°. The SNR of the incident wave is set to 30 dB.

Figure 12 shows that a channel capacity of over 220 bits/s/Hz is achieved when *r* is greater than 30 cm, which is equivalent to 22 Gbps for a bandwidth of 100 MHz. The channel capacity increases by 52 bits/s/Hz when *r* = 30 cm, compared with the case where *r* = 15 cm. Furthermore, the channel capacity remains constant after *r* = 30 cm, meaning that, considering the size of the array, *r* = 30 cm is one of the best possible solutions for obtaining the maximum channel capacity with an array of limited size. Note that the black and red curves at angles of 0° and 45° closely overlap. This is due to the fact that, as described in Figure 4, the 32 × 32 MIMO array uses different combinations of subarrays, Combination1 and Combination2, at *ϕ* = 0° and 45°, which enables the radiation gain in these directions to be enhanced with equal intensity. Consequently, we have the same level of channel capacity at these angles, which confirms that the beam steering function operates correctly, regardless of the size of the array.

**Figure 13:**
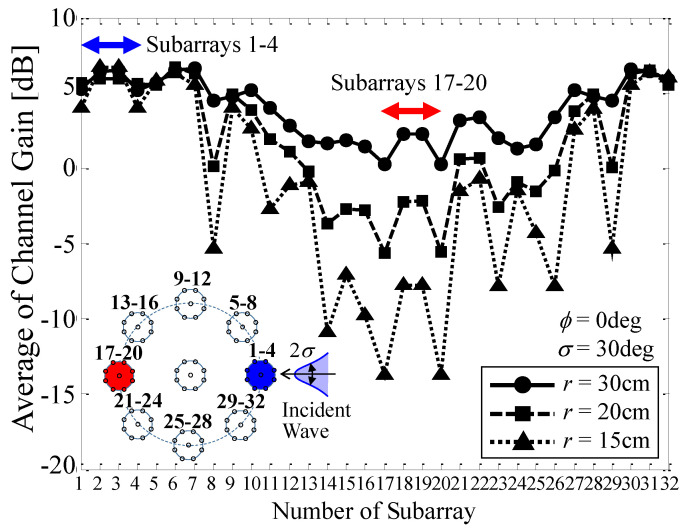
Channel gain as a function of the number of each subarray.

To understand the mechanism for the radius-channel capacity relationship more specifically, we performed an analysis of the channel gain. Figure 13 shows the channel gain as a function of the number of the subarray with the radius as a parameter when a single Gaussian wave with a standard deviation of *σ* = 30° comes from an angle of *ϕ* = 0°. The channel gain is defined as the ratio of the received power of the signal at each subarray to that of a single isotropic antenna. Thus, a channel gain exceeding 2.15 dB means that the subarray has more gain than an ordinary dipole antenna. Note that we obtained the channel gain in such a way that the average operation is performed with respect to all 32 incident waves.

In Figure 13, we calculated three cases; *r* = 30 cm, *r* = 20 cm, and *r* = 15 cm. The inset in the figure shows the geometrical relationship between the array structure and the incident wave. The blue and red subarrays indicate that these subarrays, Subarrays 1–4 and Subarrays 17–20, are located in the forward and backward directions with respect to the incident wave, as shown in the inset. The blue and red arrows included in the upper part of Figure 13 indicate the locations of Subarrays 1–4 and Subarrays 17–20 on the lateral axis.

Figure 13 shows that, regardless of the radius, Subarrays 1–4, which face the incident wave, provide a large channel gain of more than 2.15 dB, meaning that these subarrays operate more effectively than a conventional dipole antenna. On the other hand, for Subarrays 17–20, the channel gains exhibit a significant reduction as the radius decreases. In particular, when *r* = 15 cm the channel gain is reduced to −10 dB, implying that these subarrays do not yield a useful subchannel for MIMO transmission. This phenomenon for the reduction in channel gain can also be understood from the radiation patterns, as described in Figure 11. It is concluded from these results that the reduction in channel capacity with decreasing radius *r*, as described in Figure 12, is caused by a decrease in the channel gains of the subarrays, particularly that situated at the back with respect to the incident wave, as illustrated in Figure 13.

**Figure 14:**
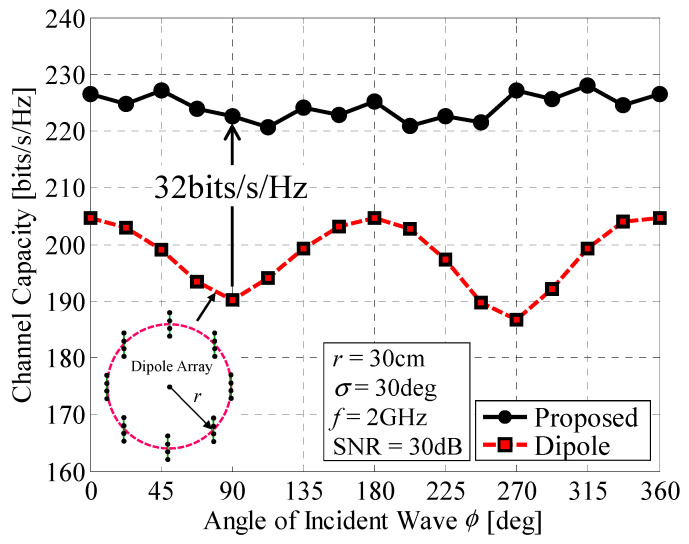
Channel capacity as a function of the angle of the incident wave.

Figure 14 shows the channel capacity of the 32 × 32 MIMO array as a function of the angle *ϕ* when *r* = 30 cm. The angular spread *σ* and SNR were set to 30° and 30 dB, respectively. For comparison, the channel capacity of a 32 × 32 MIMO dipole array is included in the graph. The dipole array comprises eight groups of four-element dipole linear arrays with a spacing of 3 cm arranged at equal 45-degree angles aligned along the *y*-axis, situated at the same locations as Subarrays 1–4 to 29–32, as indicated by the inset in the figure. As shown in Figure 14, when the proposed antenna is used, the channel capacity remains constant regardless of the angle of the incident wave. In contrast, in the case of the dipole array antenna, more than 32-bits/s/Hz degradation in the channel capacity is observed when the angle of the incident wave is 90° or 270°, in which the dipole array is parallel to the incident wave. The dipole array gives a channel capacity of less than 205 bits/s/Hz because of low SNR or high correlation. In contrast, the 32 × 32 MIMO array achieves a constant channel capacity of 220 bits/s/Hz regardless of the direction of the incoming wave owing to the full-azimuth beam steering function.

### 5.3. Antenna–Propagation Mutual Interactions

As mentioned in the introduction, the MIMO antenna for a connected car system, implemented in the framework for the fifth and sixth generation (5G and 6G) mobile communications, is anticipated to be used in a system where the distance between the base station and the mobile station is small, compared with the conventional macrocell counterpart. In a small cell system, there are two significant factors to be considered that affect the channel capacity of a MIMO system; the signal-to-noise power ratio (SNR) and the spatial angular power spread of the incoming waves. The interaction between these two physical quantities is complex and there might be a tradeoff relationship because, for example, when a mobile station is situated in close proximity to a base station the received power of the mobile station increases due to the large SNR but the angular power spread of the received signal decreases owing to the line-of-sight (LOS) property of the radio propagation mechanism.

**Figure 15:**
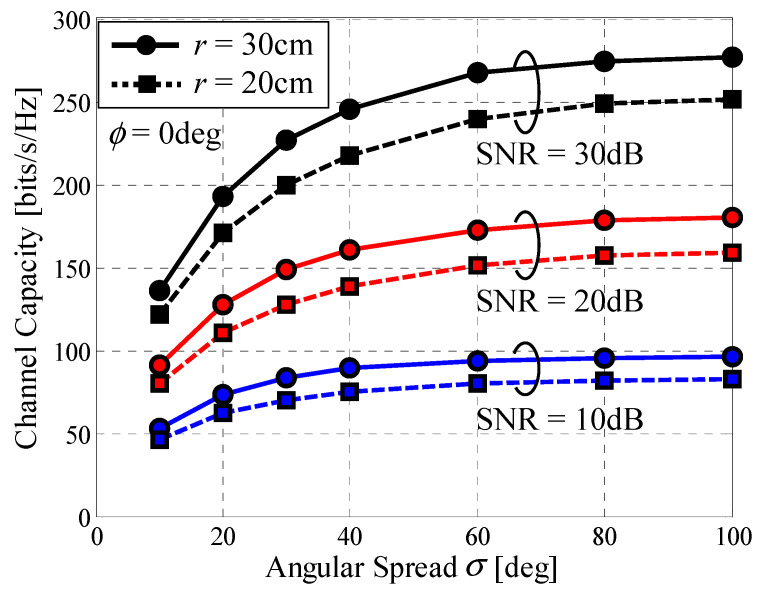
Channel capacity vs. the angular power spread.

On the basis of the above-mentioned background, we carried out a Monte Carlo simulation to investigate how the developed 32 × 32 MIMO array performs in a propagation environment where forthcoming 5G and 6G connected car systems are anticipated to be used. Figure 15 shows the channel capacity as a function of the angular power spread of an incident wave with the SNR as a parameter. In Figure 15, the solid lines denote the cases where the radius of the array *r* is 30 cm and the dashed lines represent the cases of *r* = 20 cm. We assume a single incident wave coming from *ϕ* = 0° along the *x*-axis.

It can be seen from Figure 15 that the 32 × 32 MIMO array achieves a maximum channel capacity of 277 bits/s/Hz when SNR = 30 dB, *r* = 30 cm, and *σ* = 100°. Moreover, Figure 15 shows that the channel capacity decreases significantly as the angular power spread of the incoming wave decreases. Specifically, when SNR = 30 dB and *r* = 30 cm the channel capacity is 268 bits/s/Hz in the case of *σ* = 60°. In contrast, the channel capacity reduces to 135 bits/s/Hz in the case of *σ* = 10°, which is equivalent to a 50% reduction. In [10], the channel model parameters for various cellular systems are summarized, and the angular spread at the mobile station is found to vary between 30° and 80° (see Table 2 in [10]). Judging from this fact, Figure 15 indicates that the developed antenna has good performance in situations encountered in typical application scenarios.

To clarify the mechanism responsible for the observations described in Figure 15, the channel gain and correlation characteristics were investigated.

**Figure 16:**
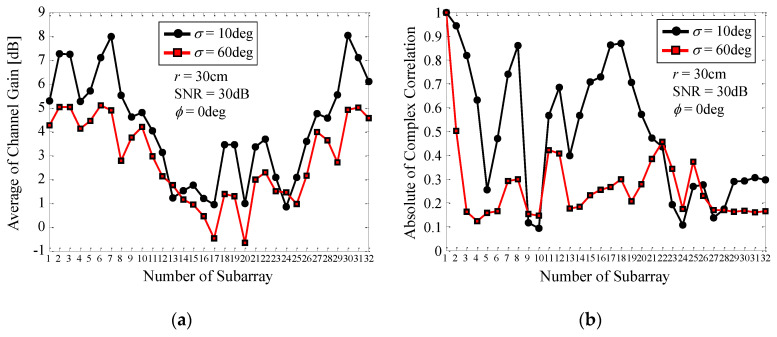
Channel gain and correlation characteristics of each subarray. (**a**) Channel gain. (**b**) Correlation.

Figure 16 shows the channel gain and correlation coefficient of each subarray of the 32 × 32 MIMO array as a function of the number of the subarray. The black lines with round symbols denote the case where *σ* = 10°, whereas the red lines with square symbols represent the case where *σ* = 60°. The correlation coefficient is defined as the absolute value of the complex correlation coefficient between Subarray1 and the other subarrays, defined as *ρ_a_* in Equation (10). Note that we obtained the correlation coefficient in such a way that the average operation is performed with respect to all 32 incident waves. As described in Figure 6, and Equations (3) and (4) in Section 4, the incident waves from the base station are assumed to be plane waves, i.e., far-field assumption, and thus the channel model does not include the effects of the distance between the base and mobile stations on the correlation.

It can be seen from Figure 16a that a channel gain of more than 2 dB is obtained for the subarrays numbered 1–8 and 29–32 for both *σ* = 10° and 60°. Particularly, the channel gain for *σ* = 10° exceeds 4 dB and is larger than that for *σ* = 60°. This is due to the fact that when *σ* = 10° the peak gain at *ϕ* = 0° results in a large channel gain due to in-phase excitation of the array, as depicted in Figure 11. In contrast, the channel gain for the subarrays numbered 13–24 is small in magnitude because of the shadowing effects caused by the subarrays located in front of them. Figure 16b shows that the correlation coefficients for *σ* = 10° are larger than those for *σ* = 60°. Especially, the correlations between Subarrays 1 and 2, 1 and 3, and 1 and 4, which belong to the same 4 × 4 MIMO array group, are more than 0.6. In contrast, when *σ* = 60° the correlation is less than 0.5 over the entire array.

**Figure 17:**
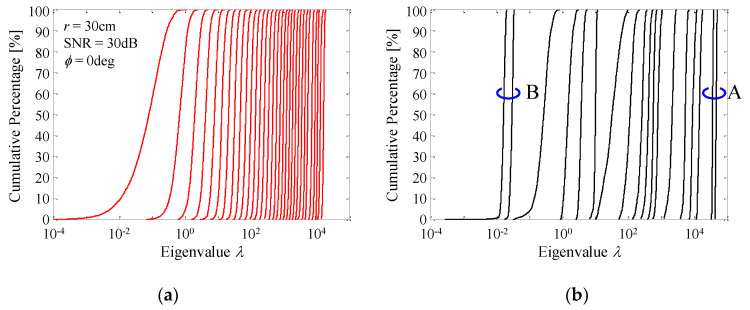
CDF characteristics of eigenvalues. (**a**) *σ* = 60°. (**b**) *σ* = 10°.

The changes in the channel gain and correlation coefficients mentioned above result in a variety of distributions of eigenvalues. Figure 17a,b show the cumulative distribution functions (CDF) of the eigenvalues when *σ* = 60° and *σ* = 10°. The eigenvalues are obtained from Equation (14) through an SVD operation.

In Figure 17a, the presence of 32 densely distributed eigenvalues in the graph means that there are 32 effective subchannels for MIMO transmission. This indicates that the channel matrix defined by Equation (8) has a full-rank property corresponding to the number of subarrays because all the subarrays can receive signals from the communication target owing to the beam steering function of the proposed antenna. This feature is quite different from a conventional circular array antenna with a number of patch antennas arranged in a cylindrical manner [7,8], as described in the introduction. Consequently, the developed antenna operates normally and effectively as a 32-element MIMO array antenna.

In contrast, the sparsely distributed 18 eigenvalues, shown in Figure 17b, indicate that the developed array works as a MIMO antenna with a small number of elements. More specifically, a large channel gain in the case of *σ* = 10° provides large first and second eigenvalues, as indicated by the symbol “A” on the right side of Figure 17b. Moreover, a high correlation coefficient for *σ* = 10° creates small eigenvalues distributed on the left side of Figure 17b, as indicated by the symbol “B”. Eventually, when *σ* = 10° these two observations result in a small channel capacity, as described in Figure 15.

From the extensive analytical investigations performed in this paper, we conclude that a remarkably high channel capacity of more than 200 bits/s/Hz achieved by the beam steering 32 × 32 MIMO array—as depicted in Figure 12, Figure 14, and Figure 15—can be attributed to two significant factors; the improved directivity due to in-phase excitation of the array and the low correlation between subarrays caused by the orthogonal alignment of the array with respect to the incident waves.

## 6. Experimental Verification

To confirm the validity of the proposed antenna, this section presents experiments carried out to evaluate the 4 × 4 and 32 × 32 MIMO arrays. We performed two types of measurements; radiation pattern measurements in an anechoic chamber and over-the-air (OTA) testing using a two-dimensional fading emulator in a cluster propagation environment [26].

### 6.1. Impedance and Radiation Measurements

**Figure 18:**
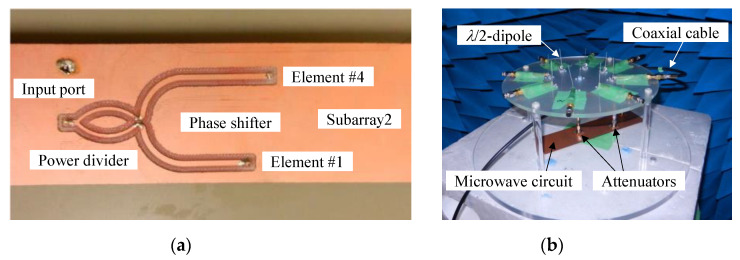
Radiation pattern measurement setup of a 4 × 4 MIMO array in an anechoic chamber. (**a**) Fabricated microwave circuit. (**b**) Prototype of a 4 × 4 MIMO array.

Figure 18 shows the setup for measuring the radiation pattern of a 4 × 4 MIMO array in an anechoic chamber [15]. The signal processing unit was designed using the circuit topology depicted in Figure 8a, including a phase shifter and a signal combiner. Figure 18a shows a fabricated microwave circuit for the weight functions of Subarry2 (*w*_1_ and *w*_4_) using a double-sided printed circuit board with a thickness of 1 mm and a relative permittivity of 4.2 (FR4). The phase shift of the received signal for each element was realized using different lengths of microstrip transmission lines. The 30° phase difference between #1 and #4 is equivalent to a difference in length of 7.6 mm. The phase difference of the produced network was 34°, indicating that the phase difference was achieved. Furthermore, to get the correct weight function, the difference in power loss between the microstrip lines, which was 0.21 dB, was considered.

An attenuator was used for realizing the weight function. Since *w*_1_ = 1.0 and *w*_4_ = 0.38 for Subarray2, as listed in Table 1, the received signal of #4 had to be synthesized by attenuating 8.40 dB compared to that of #1. Since the power loss for #4 due to the microstrip line was 0.21 dB larger than that for #1, a power loss of 8.19 dB had to be realized by the attenuator. 0 dB and 8 dB attenuators were connected to the terminals of #1 and #4 of the fabricated microwave circuit, respectively. The 0 dB attenuator was used for realizing the retention of the phase of the received signals of #1 and #4. Hence, the difference in the received power between #1 and #4 was 8.50 dB, indicating that the weight function had been achieved.

The radiation pattern of a 4 × 4 MIMO array antenna was measured in an anechoic chamber. Figure 18b shows the measurement setup of a 4 × 4 MIMO antenna, which comprises eight half-wavelength dipole antennas designed at 2 GHz. Two attenuators and the fabricated microwave circuit were located under the antenna and connected to the antenna using coaxial cables. As shown in Figure 18b, the prototype of a 4 × 4 MIMO antenna was mounted on a turntable for sampling signals of the radiation patterns at azimuth angles in the *xy*-plane using a vector network analyzer.

**Figure 19:**
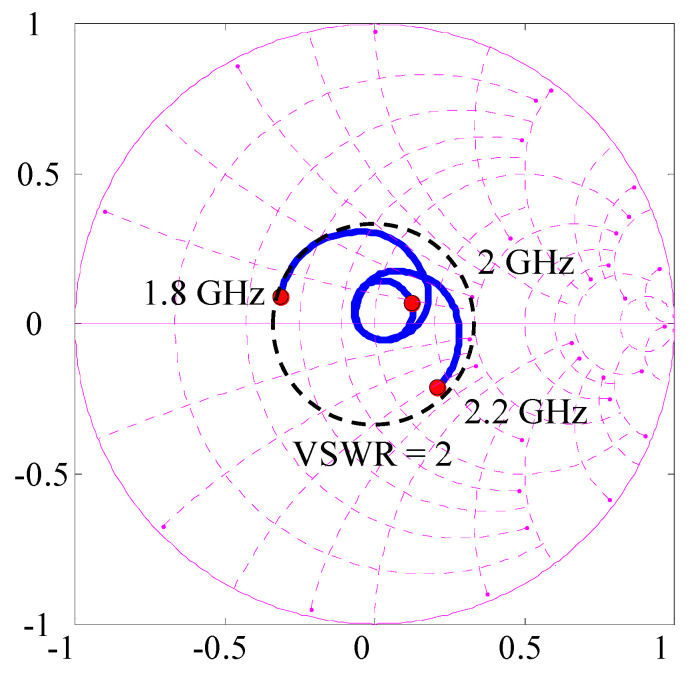
Measured impedance of Subarray2.

Figure 19 exhibits the impedance characteristics of Subarray2 drawn on a Smith chart measured at the input port of the microwave circuit, as indicated in Figure 18a, using a network analyzer [15]. In Figure 19, the black broken circle represents the impedance locus corresponding to a voltage standing wave ratio (VSWR) of 2. It can be seen from Figure 19 that a good matching condition with a VSWR of less than 2 was achieved in the frequencies between 1.8 GHz and 2.2 GHz, where existing MIMO-based cellular systems are being operated.

**Figure 20:**
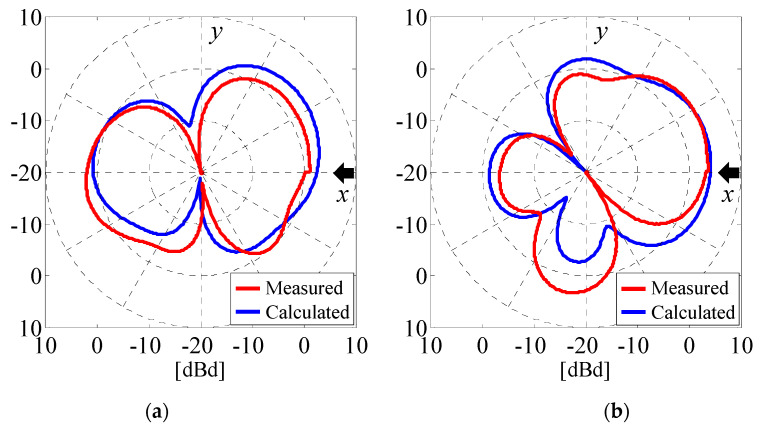
Measured and calculated results of radiation patterns. (**a**) Subarray1. (**b**) Subarray2.

Figure 20 presents the measured and calculated *θ*-polarized radiation patterns of Subarray1 and Subarray2 in the horizontal (*xy*) plane when the angle of the incident wave is 0° [27]. In the measurement, we performed a calibration to compensate for the power loss. The red line indicates the measured results, while the blue line represents the calculated results. The frequency for the measurements was 2 GHz.

As shown in Figure 20, there are some appreciable discrepancies between the measured and calculated results, particularly in the sidelobe and backlobe angular regions. A possible cause of these discrepancies is the electromagnetic distortion arising from coaxial feed cables connected between the dipole elements and the microwave circuit, as shown in Figure 18b. Nevertheless, good agreement between the measured results and the analytical outcome can be observed in the direction of the *x*-axis indicated by the black arrows, confirming that the weight functions and the phase shift values listed in Table 1 work well according to the angle of the incident wave.

### 6.2. Over-the-Air Testing

**Figure 21:**
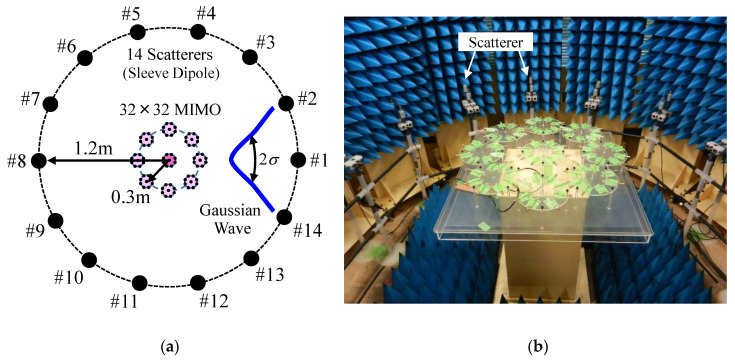
OTA testing of a 32 × 32 daisy chain MIMO antenna. (**a**) Two-dimensional fading emulator. (**b**) External view.

The channel capacity was measured using a two-dimensional fading emulator [26], as illustrated in Figure 21.

In [28], the appropriate number of probe antennas for a fading emulator was investigated theoretically. The results show that 11–15 scatterers need to be implemented for achieving a good multi-cluster propagation environment. On the basis of this knowledge, we have developed a fading emulator with 14 scatterers.

Fourteen scatterers with equal angular intervals were arranged on a circle with a radius of 1.2 m. The scatterers comprise vertically polarized half-wavelength sleeve dipole antennas. The beam steering MIMO array antenna is located at the center of the emulator, as shown in Figure 21. This fading emulator has the function of an operating algorithm on the basis of the same formulation described in Section 4. To change the angle of the incident wave from 0° to 360°, the beam steering array was mounted on a turntable to rotate at 45° steps in azimuth while maintaining a power spectrum with a fixed Gaussian distribution generated on the scatterers, as depicted by the blue curve in Figure 21a. The experiments were performed in a cluster propagation environment, which had an angular power spectrum with a Gaussian distribution with an incident wave angle of *µ* = 0° and a standard deviation of *σ* = 30°. The frequency was 2 GHz. The SNR was set to 30 dB and 40 dB.

**Figure 22:**
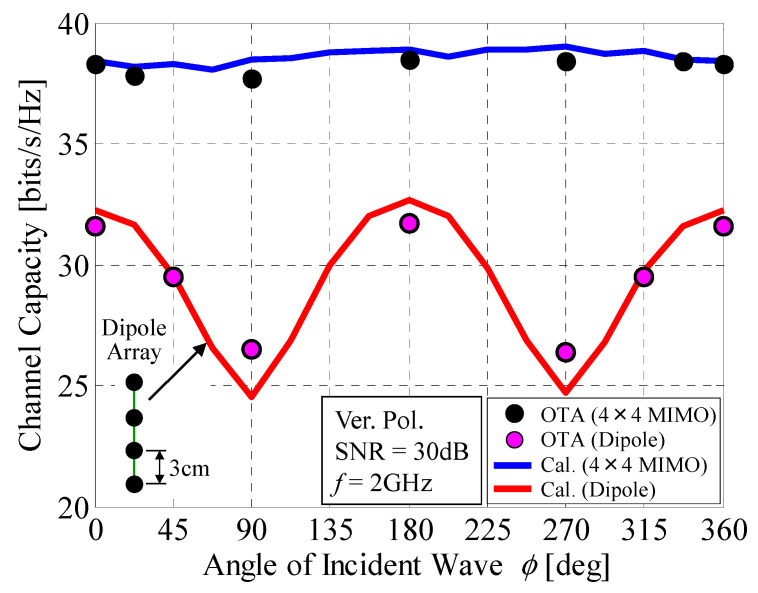
Channel capacity of a 4 × 4 MIMO array antenna measured by OTA testing.

Figure 22 shows the measured channel capacity of the 4 × 4 MIMO antenna, as illustrated in Figure 18b, as a function of the angle of the incident wave. Half-wavelength dipole antennas were used for the array elements. In the experiments, using the microwave circuit in Figure 18a, we manually switched the RF ports to set the amplitude and phase appropriate for each subarray, in accordance with Table 1. The development of a switching network, as depicted in Figure 5, for conducting time-independent measurements is a subject for our future studies.

In Figure 22, The black round symbols denote the measured results of the 4 × 4 MIMO antenna, whereas the blue curve represents the analytical results using Monte Carlo simulations. The red curve depicts the measured results of a four-element linear dipole array comprising vertically polarized half-wavelength dipole antennas with a spacing of 3 cm, as denoted by the inset in Figure 22, in which the entire array size is the same as the 4 × 4 MIMO antenna. Figure 22 shows that there is good agreement between the measured and calculated results for the 4 × 4 MIMO antenna. Furthermore, the proposed antenna maintains a constant transmission rate over 360 degrees of azimuthal angle, whereas the dipole array yields heavy fluctuations with deep nulls at angles of 90° and 270°, which is similar to the result for the 32 × 32 MIMO array antenna described in Figure 14.

**Figure 23:**
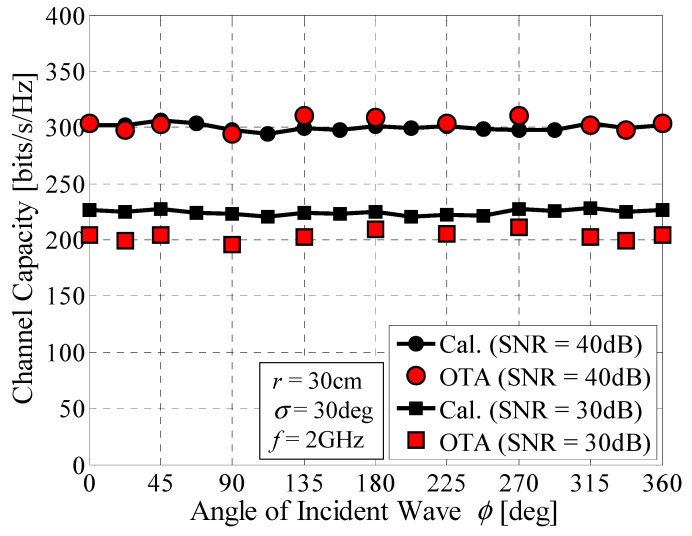
Channel capacity of a 32 × 32 MIMO array antenna measured by OTA testing.

Figure 23 shows the channel capacity of a prototype of the 32 × 32 daisy chain MIMO antenna as a function of the angle of the incident wave [15]. The radius of the array was set to *r* = 30 cm. The SNR was set to 30 dB and 40 dB. The red round and square symbols denote the measured results. The black curves represent the analytical results from Monte Carlo simulations. Figure 23 shows that there is good agreement between the measured and calculated results. Furthermore, the proposed antenna can maintain a constant transmission rate over 360 degrees in azimuth. Interestingly, the red round and square symbols corresponding to angles of 22.5° and 337.5° are situated at the borders between two adjacent sectors, as illustrated in Figure 4. These measured data do not show appreciable changes, compared with 0° and 360°, indicating that the proposed antenna works well, even on the borders of the sectors, owing to the good beam steering ability of the array.

In Section 5.3, the channel gain and correlation were obtained from a theoretical investigation using Monte Carlo simulation, as shown in Figure 16. In this section, to investigate the mechanism for achieving a large channel capacity in an empirical way, we took an experimental approach by means of OTA testing to evaluate the channel gain and correlation.

**Figure 24:**
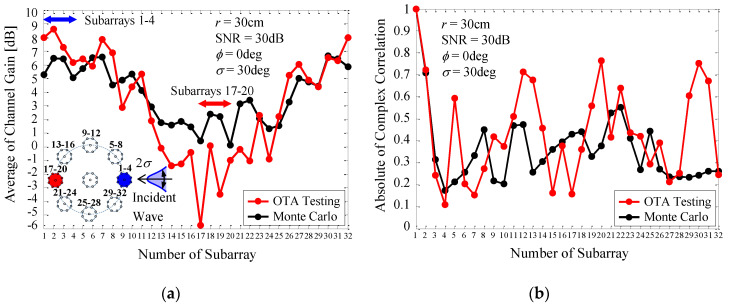
Channel gain and correlation of a 32 × 32 MIMO array measured by OTA testing. (**a**) Channel gain. (**b**) Correlation.

Figure 24 shows the channel gain and correlation coefficient of each subarray of the 32 × 32 MIMO array as a function of the number of the subarray [15]. The black curves indicate the analytical results using Monte Carlo simulation, while the red curves indicate the measured results through OTA testing, depicted in Figure 21. The blue and red subarrays, indicated by the inset in Figure 24a, represent that these subarrays. Subarrays 1–4 and Subarrays 17–20 are located in the forward and backward directions with respect to the incident wave. The blue and red arrows included in the upper part of Figure 24a indicate the locations of Subarrays 1–4 and Subarrays 17–20 on the lateral axis.

Figure 24a shows that Subarrays 1–4, which face the incident wave, provide a large channel gain of more than 2.15 dB, meaning that these subarrays operate more effectively than a conventional dipole antenna. On the other hand, for Subarrays 17–20, the channel gain is significantly reduced to less than 0 dB. This implies that these subarrays do not contribute to the formation of subchannels available for MIMO transmission. It is worth noting that, in Figure 24a, there is an appreciable discrepancy between the OTA measurements and the analytical outcomes. This is due to the fact that Subarrays 1–4, 5–8, and 29–32, located in the forward direction with respect to the incident wave, receive strong signals from the scatterers because of the small distance between these subarrays and the scatterers emitting a cluster wave, as shown in Figure 21a. In contrast, Subarrays 13–16, 17–20, and 21–24, located at the back with respect to the direction of the incident wave, receive weak signals because of the large distance between these subarrays and the scatterers.

Figure 24b shows that, in the OTA measurements, the correlation coefficient increases in a periodic manner, which is not observed in the analytical outcome from the Monte Carlo simulations. A possible cause of this phenomenon is the insufficient orthogonality between the incident waves coming from the scatterers, because, in the current experiment, the number of scatterers in the emulator is 14, which is considerably smaller than the number of subarrays, i.e., 32. Nevertheless, it can be seen from Figure 24b that except for the correlation between those particular subarray numbers showing a periodic increase, the correlation coefficients are less than 0.5 over the entire array because of the orthogonal alignment between the MIMO array and the incident waves.

## 7. Conclusions

This paper presents a 32 × 32 MIMO antenna system with the ability to perform full-azimuth beam steering. Analyses revealed that the proposed antenna system can provide a maximum channel capacity of 277 bits/s/Hz at an SNR of 30 dB when the radius of the array is 30 cm, which is equivalent to 27.7 Gbps with a bandwidth of 100 MHz. It was clarified that this remarkably high channel capacity is due to two significant factors; the improved directivity due to the optimum in-phase excitation and the low correlation between the subarrays due to the orthogonal alignment of the array with respect to the incident waves. We further performed an experimental validation of the channel capacity using over-the-air (OTA) testing. The results show that the proposed antenna can maintain a constant channel capacity over 360 degrees in azimuth.

The developed technology can be applied to the achievement of a 64 × 64 MIMO array [29] and a 128 × 128 MIMO array [18] in pursuit of much larger transmission rates in the 5 GHz frequency band. Thereby, the daisy chain array structure proposed in this paper provides a strong solution for realizing a much higher order MIMO array antenna at higher frequencies, such as in the 5.9 GHz band, where the emerging vehicle-to-everything (V2X) and cellular-V2X (C-V2X) technologies are anticipated to be operated for future connected cars [30,31,32].

## Figures and Tables

**Table 1:** Excitation conditions of each element.

	Frontward for Incident Wave	Backward for Incident Wave
Subarray1	#2 1 V, −160°	#3 0.72 V, 0°
Subarray2	#1 1 V, −330°	#4 0.38 V, 0°
Subarray3	#8 1 V, −330°	#5 0.38 V, 0°
Subarray4	#7 1 V, −160°	#6 0.72 V, 0°

**Table 2:** Analytical conditions.

Frequency	2000 MHz
Number of elements (*M*, *N*)	*M* = *N* = 32
Number of clusters (*Q*_c_)	1
Number of scatterers (*K*_m_)	30
Initial phase for scatterers	Random
Traveling distance (*d*) (dependent on angular spread)	1000λ for *σ* = 10° 100λ for σ = 100°
Number of samples (*S*)	5000
Sampling interval (Δ*d* = *d*/*S*) (dependent on angular spread)	0.2λ for *σ* = 10° 0.02λ for *σ* = 100°
Moving direction (*ϕ_V_*)	5°
XPR	50 dB (Vertical Pol.)
Antenna element	Half-wavelength dipole
Method of EM analysis	Method of moments
